# CD133 expression in chemo-resistant Ewing sarcoma cells

**DOI:** 10.1186/1471-2407-10-116

**Published:** 2010-03-26

**Authors:** Xiaohua Jiang, Ynnez Gwye, Darren Russell, Christine Cao, Dorothea Douglas, Long Hung, Heinrich Kovar, Timothy J Triche, Elizabeth R Lawlor

**Affiliations:** 1 Saban Research Institute, Childrens Hospital Los Angeles, 4650 Sunset Blvd, Los Angeles, CA 90027, USA; 2 Children's Cancer Research Institute, St. Anna Kinderspital, Kinderspitalgasse 6, 1090 Vienna, Austria; 3 Departments of Pediatrics, University of Southern California, Los Angeles, CA 90089, USA; 4 Pathology, Keck School of Medicine, University of Southern California, Los Angeles, CA 90089, USA; 5 University of Michigan, 1150 West Medical Center Drive, 1200 MSRBIII/SPC 5632, Ann Arbor, MI 48109-5632, USA

## Abstract

**Background:**

Some human cancers demonstrate cellular hierarchies in which tumor-initiating cancer stem cells generate progeny cells with reduced tumorigenic potential. This cancer stem cell population is proposed to be a source of therapy-resistant and recurrent disease. Ewing sarcoma family tumors (ESFT) are highly aggressive cancers in which drug-resistant, relapsed disease remains a significant clinical problem. Recently, the cell surface protein CD133 was identified as a putative marker of tumor-initiating cells in ESFT. We evaluated ESFT tumors and cell lines to determine if high levels of CD133 are associated with drug resistance.

**Methods:**

Expression of the CD133-encoding *PROM1 *gene was determined by RT-PCR in ESFT tumors and cell lines. CD133 protein expression was assessed by western blot, FACS and/or immunostaining. Cell lines were FACS-sorted into CD133+ and CD133- fractions and proliferation, colony formation in soft agar, and *in vivo *tumorigenicity compared. Chemosensitivity was measured using MTS (3-(4,5-dimethylthiazol-2-yl)-5-(3-carboxy-methoxyphenyl)-2-(4-sulfophenyl)-2H-tetrazolium) assays.

**Results:**

*PROM1 *expression was either absent or extremely low in most tumors. However, *PROM1 *was highly over-expressed in 4 of 48 cases. Two of the 4 patients with *PROM1 *over-expressing tumors rapidly succumbed to primary drug-resistant disease and two are long-term, event-free survivors. The expression of *PROM1 *in ESFT cell lines was similarly heterogeneous. The frequency of CD133+ cells ranged from 2-99% and, with one exception, no differences in the chemoresistance or tumorigenicity of CD133+ and CD133- cell fractions were detected. Importantly, however, the STA-ET-8.2 cell line was found to retain a cellular hierarchy in which relatively chemo-resistant, tumorigenic CD133+ cells gave rise to relatively chemo-sensitive, less tumorigenic, CD133- progeny.

**Conclusions:**

Up to 10% of ESFT express high levels of *PROM1*. In some tumors and cell lines the CD133+ fraction is relatively more drug-resistant, while in others there is no apparent difference between CD133+ and CD133- cells. These studies reveal heterogeneity in *PROM1*/CD133 expression in ESFT tumors and cell lines and confirm that high levels of *PROM1 *expression are, in at least some cases, associated with chemo-resistant disease. Further studies are required to elucidate the contribution of *PROM1/*CD133 expressing cells to therapeutic resistance in a large, prospective cohort of primary ESFT.

## Background

Ewing's sarcoma family tumors (ESFT) are malignant tumors of bone and soft tissue that occur predominantly in adolescents and young adults. Histologically they appear as small round blue cell tumors and, although some tumors display evidence of neural differentiation, they more commonly lack defining morphologic features [1]. Genetically, ESFT are characterized by recurrent chromosomal translocations that result in the creation of fusion oncogenes, most commonly *EWS-FLI1 *or *EWS-ERG *[1]. For patients who present with localized tumors, aggressive multi-modal therapy results in 5-year overall survival rates of over 70% [2]. However, for patients who present with overt metastasis, who fail to respond to induction chemotherapy, or who relapse, the prognosis remains dismal.

In recent years putative tumor-initiating cancer stem cells have been isolated from human tumors (reviewed in Ref. [3]). These cells exist as a minority population within the tumor, possess the stem cell properties of self-renewal and multi-lineage differentiation capacity and are proposed to be the root cells from which tumors are derived and maintained [4-10]. Importantly, it has been shown that tumor-initiating cancer stem cells may be more resistant to standard chemo- and radiation-based therapies than bulk tumor cells [11-14]. In addition, although definitive proof of tumor-initiating populations requires study of freshly isolated primary tumor samples, the cellular hierarchy of tumorigenicity is sometimes preserved in established cancer cell lines [15].

Recently, it was reported that the cell surface glycoprotein CD133 is a marker of tumor-initiating cells in ESFT [16]. For the current study we evaluated expression of the CD133-encoding *PROM1 *gene in a large panel of ESFT tumors and cell lines to determine if high expression might be useful as a marker of drug resistance in ESFT. Our data show that *PROM1 *expression is usually extremely low in primary tumors. However, we identified in a small subset of cases (4 of 48) in which *PROM1 *was highly over-expressed. Two of 4 tumors with *PROM1 *over-expression were highly resistant to primary chemotherapy and 2 were responsive. Studies of ESFT cell lines also showed considerable heterogeneity in *PROM1 *expression and in the frequency and chemosensitivity of CD133+ and CD133- cell fractions. Importantly, however, in one cell line - STA-ET-8.2 - CD133 expression was found to be associated with enhanced tumorigenic potential and increased drug resistance. Thus, in at least some ESFT tumors and cell lines, CD133+ cells are relatively more resistant to therapy than their CD133- counterparts. The potential utility of *PROM1/*CD133 as a marker of therapeutic resistance in ESFT requires prospective evaluation in a large cohort of newly diagnosed patients.

## Methods

### Cell Lines & Tumor Specimens

ESFT cell lines were maintained and passaged as cellular monolayers in 10% fetal bovine serum-containing media as described [17,18]. STA-ET-8.2 cells were obtained from Children's Cancer Research Institute, Vienna, Austria and the remainder of cell lines from Childrens Hospital Los Angeles (CHLA), CA. Primary tumor sections and RNA were obtained from the Children's Oncology Group (COG) Biorepository in Columbus, Ohio (Cooperative Human Tissue Network - CHTN) and the CHLA tumor bank. All specimens were obtained in compliance with HIPAA regulations and following protocol review by institutional review board. Informed consent for use of tumor samples for research purposes was obtained from each subject or subject's guardian.

### Flow Cytometry & Cell Sorting

Cultured cells were trypsinized and resuspended in FcR Blocking Reagent (Miltenyi Biotec, Auburn, CA, USA) with 0.5% bovine serum albumin (Sigma, St Louis, MO, USA) in phosphate-buffered saline (PBS). Mouse anti-human CD133/2-PE (Miltenyi) monoclonal antibody was then added (1:11 dilution) and incubated for 15 min at 4°C in the dark. After two washes, labeled cells were analyzed by a FACScan flow cytometer (Becton Dickinson). A minimum of 10,000 events was collected and acquired using CellQuest software (Becton Dickinson). For flow-activated cell sorting (FACS), cells were stained for CD133/2-PE and isolated on FACS Vantage or FACS Aria instruments (BD Biosciences). Analysis was done using the Flow-Jo program (Tree Star, Ashland, OR). Positive and negative gates were determined using IgG stained and unstained controls. Isolated cells were then washed twice with PBS and plated under the same conditions as unsorted cells. For cell lines with less than 20% CD133+ cells, FACS was preceded by magnetic bead sorting (MACS) to first enrich for the CD133 population and thereby improve FACS efficiency. Cells were labeled with primary CD133/1 antibody (mouse IgG1, Miltenyi Biotec, 1 ul per million cells), magnetically labeled with rat anti-mouse IgG1 Micro beads (Miltenyi Biotec, 20 ul per 10 million cells) and separated by MACS LS column (Miltenyi Biotec) according to manufacturer's instructions. Since MACS-separated cells might be saturated with the antibody (CD133/1), it is recommended by the manufacturer to use an alternative antibody recognizing the second epitope of CD133 (CD133/2) when performing subsequent analyses of cell separation. Thus, we used CD133/2 in the FACS experiment when evaluating sorting efficiency of MACS-sorted cells.

### Reverse Transcription-Polymerase Chain Reaction (RT-PCR)

Total RNA was extracted from cells and frozen tissue sections using Qiagen columns (Qiagen, Valencia, CA). First strand complementary DNA (cDNA) was synthesized from 250-500 ng RNA using iScript Reverse Transcriptase kit (Bio-Rad, Hercules, CA). Primers for semi-quantitative RT-PCR were: *PROM1 *(CD133) sense-GACCGACTGAGACCCAACAT and antisense-TGGTTTGGCGTTGTACTCTG; *GAPDH *sense- CTTTAACTCTGGTAAAGTGG and antisense-TTTTGGCTCCCCCCTGCAAAT. Quantitative realtime RT-PCR (Q-RT-PCR) was performed using a validated TaqMan Gene Expression Assay (Applied Biosystems; Hs01009261-m1) which detects both known isoforms of *PROM1*. Assays were performed in triplicate on an Applied Biosystems 7900HT system. Average Ct values were normalized relative to expression of *GAPDH *and β *-Actin *in the same sample using the formula: % expression = 2^-ΔCt ^× 100.

### Western Blot

Whole cell lysates were analyzed by western blot using standard procedures. Primary antibodies used were: anti-CD133 (1:500)(Santa Cruz Biotech, Santa Cruz, CA); anti- Bax (1:1000) (BD Pharmingen, San Jose, CA); anti-Bcl-XL(1:200)(Santa Cruz Biotech, Santa Cruz, CA); anti-Bcl2(1:200)(Santa Cruz Biotech, Santa Cruz, CA); anti-survivin 1 (1: 1000)(Novus Biologicals, Littleton, CO); anti-ABCG2 (1:500)(Abcam, Cambridge, MA) and anti-β-actin (1:1000)(Santa Cruz Biotech, Santa Cruz, CA). Immunostaining cells cultured on chamber slides were rinsed in PBS and fixed in ice cold acetone for 5 min. For frozen tissue samples tumor tissue was fresh-frozen in Tissue-Tek O.C.T. compound (Sakura Finetechnical, Tokyo, Japan), cryosectioned at 10 um then fixed in acetone for 10 min at -20°C. To detect CD133, tumor sections and chamber slides were pre-incubated with 10% normal Donkey serum, then hybridized with anti-CD133 antibody (rabbit polyclonal IgG; 1: 200; Abcam, Cambridge, MA) for 1 hour at room temperature. The primary antibodies were detected with Cy3 conjugated Donkey anti-rabbit IgG, (1: 500; Jackson Immuno West Grove, PA). Sections were counterstained with DAPI, viewed with a Leica DM RXA Upright Fluorescence Microscope and photographed using a SKY camera on the system (Applied Spectral Imaging, Inc., Carlsbad, CA).

### Cell Growth and Viability Assay

FACS-sorted cells were plated in triplicate wells at a density of 3 × 10^4 ^cells/35 mm dish and total cell counts and cell viability determined using a ViCell XR cell counter (Beckman Coulter) on days 0, 1, 3, 5, 7 and 14. Cell growth and viability following drug treatment was assessed using the CellTiter-GIo Luminescent Cell Viability Assay (Promega, Madison, MI). Cells were plated at a density of 5 × 10^3 ^cells/well in 96 flat-bottomed plates, allowed to attach overnight, and then chemotherapeutic agents added at increasing concentrations. Survival of cells was assessed 24-96 hrs post-treatment with Doxorubicin, Etoposide and/or Vincristine as individual agents or in combination. (Calbiochem, San Diego, CA).

### Colony and Sphere Formation Assays

For soft agar colony formation assays cells were plated as single cell suspensions in 0.35% noble agar as previously described [19]. Cells were maintained at 37°C in a humidified incubator for 2-4 weeks and macroscopic colony formation assessed. To assess sphere-forming ability in serum and non-serum containing media in nonadherent conditions, single FACS-sorted CD133+ or CD133- cells were seeded into low attachment 96-well plates. Visual inspection was performed the day after the initial plating to confirm that each contained a single cell. After 3-4 weeks, spheres that contained > 50 cells were counted.

### Xenograft Assays

Nonobese diabetic (NOD)-severe combined immunodeficiency (SCID) mice (Charles River Laboratories) were injected with 5 × 10^6 ^ESFT parent or CD133 FACS-sorted cells. Tumor growth was monitored over time and the frequency of tumor formation compared between sorted and unsorted cells. Studies were carried out with the assurance of the Institutional Animal Care and Usage Committee.

### Statistical Analysis

All assays were repeated at least 3 times and values in the figures and text are reported as means ± SD. Statistically significant differences (p.0.05) between mean values was determined by Student's t test.

## Results

### Increased expression of *PROM1 *and CD133+ cell frequency in a subset of primary ESFT

**Figure 1:**
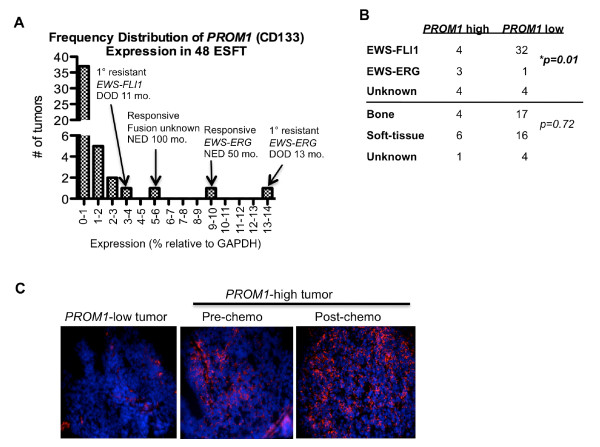
***PROM1 *is highly expressed by some primary tumors**. (A): *PROM1 *expression was assessed by quantitative RT-PCR in 48 primary ESFT specimens. Levels of expression (normalized relative to *GAPDH *in the same sample) were < 1% in 37 tumors, 1-2% in 5 tumors, 2-3% in 2 tumors, and > 3% in 4 tumors (marked with arrows). Median expression of all 48 tumors was 0.06%. Similar expression data were generated when Ct values were normalized to expression of *ACTIN *instead of *GAPDH *(not shown). Two of 4 high-expressing cases demonstrated primary drug resistance resulting in early death. NED: no evidence of disease at last follow-up; DOD: dead of disease. (B): Translocation and tissue of origin data for the 48 tumors evaluated in (A). *EWS-ERG+ *fusions were significantly over-represented among the 11 *PROM1 *expressing tumors (p = 0.01, Fisher's exact test). (C): Frozen tumor sections obtained from a *PROM1*-negative and a *PROM1-*over-expressing tumor (highest expressing tumor from (A)) were stained for CD133. Only rare CD133+ cells were detected in the *PROM1*-negative tumor. In contrast, large numbers of CD133+ cells were detected in the chemoresistant *PROM1-*over-expressing tumor and the number of CD133+ cells increased post-therapy.

CD133+ cells were shown to be extremely rare in a study of 8 primary ESFT [16]. To define the relative levels of expression of the CD133-encoding *PROM1 *gene in a larger cohort of ESFT we evaluated 48 tumor RNA samples by quantitative RT-PCR. As shown, consistent with a low frequency of CD133+ cells, *PROM1 *expression was extremely low relative to housekeeping gene expression in most tumors (Fig 1A). In contrast, however, the *PROM1 *transcript was readily amplified from 11 tumors and in 4 tumors expression was at least 40-fold greater than the median (Fig 1A, cases marked with arrows). Data were equivalent whether normalized to *GAPDH *or *β-Actin *(not shown). Translocation and tissue of origin data were available for 40 and 43 of the 48 tumors, respectively. As expected, the majority of tumors expressed an *EWS-FLI1 *fusion; however, there was a significant over-representation of *EWS-ERG *fusions among the 11 *PROM1 *positive tumors (Fig 1B). Notably, two of the four patients with *PROM1 *over-expressing tumors had highly drug-resistant disease and died 11 and 13 months from diagnosis (one with an *EWS-FLI1 *and one with an *EWS-ERG *tumor; Fig 1A).

To determine if over-expression of the *PROM1 *transcript is indicative of an increased frequency of CD133+ cells we obtained fresh-frozen tumor sections from a *PROM1 *negative and a *PROM1 *positive case. In keeping with the relative levels of transcript expression, only rare CD133+ cells were detected in the *PROM1 *low case while large numbers of CD133+ cells were apparent in the *PROM1 *over-expressing tumor (Fig 1B). Significantly, this *PROM1-*high, *EWS-ERG*+ bone tumor was highly drug resistant and the level of *PROM1 *(data not shown) and frequency of CD133+ cells increased further following induction chemotherapy (Fig 1C) suggesting that the CD133+ cells were relatively more chemo-resistant than their CD133- counterparts (Fig 1B).

Together these data demonstrate that although CD133+ cells are usually infrequent in ESFT, in a subset of cases large numbers of CD133+ cells can be identified. Moreover, this increased frequency of CD133+ cells is reflected by increased expression of *PROM1*. Importantly, 2 of 4 *PROM1 *over-expressing tumors in this cohort were highly resistant to induction chemotherapy suggesting that, in at least some cases of primary ESFT, CD133 may be useful as a marker of chemo-resistant disease.

### CD133 expression in ESFT cell lines is highly variable and cell line-specific

**Figure 2:**
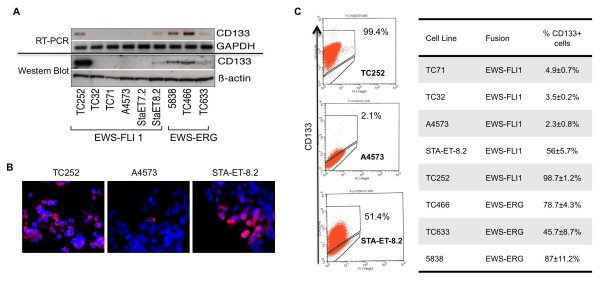
**CD133 expression in ESFT cell lines is highly variable**. (A): Expression of CD133 in nine ESFT cell lines was examined by RT-PCR and western blot analysis. The variability of protein expression in ESFT cell lines correlates with transcript expression. (B): Immunofluorescent staining of CD133 in three ESFT cell lines shows wide variation in the frequency of CD133+ cells. (C): Flow cytometric analysis of ESFT cell lines confirms widely variable frequencies of CD133+ cells in established cultures. Primary FACS plots are shown in the left panel and summary data from three independent experiments is shown in the right panel for eight different ESFT cell lines.

Although tumor-initiating cancer stem cells are definitively isolated from primary tumors [20], some cancer cell lines retain a cellular hierarchy thus permitting prospective isolation of stem cell populations from cultured cells [15]. To define the frequency of CD133+ cells in ESFT cell lines we evaluated *PROM1 *and CD133 expression in 9 well-established lines by RT-PCR and western blot, respectively. *PROM1/*CD133 expression was found to be variable but high levels were detectable in two of six EWS-FLI1+ cell lines and in all three EWS-ERG+ cell lines (Fig 2A). To establish if differing levels of expression were due to overall differences among all cells or a result of different frequencies of CD133+ cells, we used immunostaining and flow cytometry to directly assess the prevalence of CD133+ cells in each culture. The antibody we used for these studies is specific for the AC133 epitope of CD133 that is present on progenitor cells [21]. First, immunocytochemical staining revealed that while nearly all TC252 cells exhibit dense cell surface expression of CD133, STA-ET-8.2 and A4573 display a heterogeneous mixture of CD133+ and CD133- cells (Fig 2B). These data are consistent with the relative levels of overall CD133 transcript and protein detected in the three cell lines (Fig 2A). Next, we used flow cytometry to more precisely define the proportion of CD133+ vs. CD133- cells. Interestingly, although CD133+ cells were identified in all the cultures, the frequency ranged from only 2% of A4573 cells to nearly 100% of TC252 cells (Fig 2C). Together, these data demonstrate that, like primary tumors, the frequency of CD133+ cells in established ESFT cell lines varies widely and that expression is, in general, higher in *EWS-ERG *than *EWS-FLI1 *cells.

### CD133+ STA-ET-8.2 cells display enhanced proliferation *in vitro*

**Figure 3:**
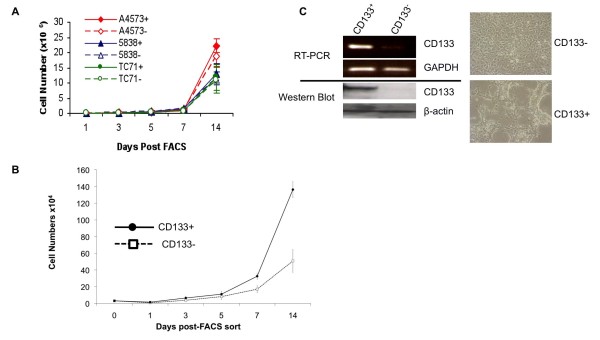
**CD133+ STA-ET-8.2 cells show enhanced growth characteristics**. (A): Direct cell counting of CD133+ and CD133- from A4573, 5838 and TC71 cultures shows no difference in growth rate between CD133+ and CD133- populations. (B): In contrast to the cell lines shown in (A), CD133+ STA-ET-8.2 cells grow faster than their CD133- counterparts. Data presented are mean ± s.d. of three independent experiments. (C): Differential expression of CD133 is confirmed in FACS-sorted STA-ET-8.2 cells (left panel). CD133+ and CD133- cells show different growth patterns with CD133+ cells demonstrating preferential growth in aggregate cell clusters, whereas CD133- cells grow as cellular monolayers (right panel).

To establish if a CD133-defined cellular hierarchy exists in ESFT cell lines we sorted four different lines into CD133+ and CD133- fractions. Growth and morphology of the sorted cells was monitored over time in several independent experiments and differential expression of CD133 expression in the sorted populations was confirmed at the start and end of each experiment by RT-PCR and/or flow cytometry (not shown). Intriguingly, although there was no difference in growth rate or morphologic appearance between CD133+ and CD133- cells in A4573, TC71 or 5838 cell lines (Fig 3A), CD133+ STA-ET-8.2 cells consistently grew faster than their CD133- counterparts (Fig 3B). In addition, we observed a marked change in cellular morphology between CD133+ cells and CD133- cells. Whereas CD133- cells grew as uniform, evenly distributed adherent monolayers, CD133+ STA-ET-8.2 cells preferentially grew in aggregate clusters of cells (Fig 3C). Together these data suggested that CD133+ STA-ET-8.2 cells might be enriched for tumor-initiating cancer stem cells.

### CD133+ STA-ET-8.2 cells form tumor spheres and produce CD133+ and CD133- progeny

**Figure 4:**
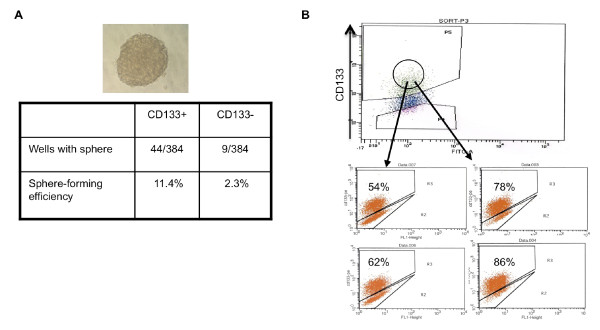
**FACS-sorted CD133+ STA-ET-8.2 cells from spheres in non-adherent culture conditions**. (A): STA-ET-8.2 cells were isolated by FACS using anti-CD133 antibody followed by plating at single cell dilution in 96 well plates. After 3-4 weeks in culture, individual spheres of > 50 cells (top image) were apparent in 11.4% of wells seed with a single CD133+ cell whereas sphere-forming efficiency from CD133- cells was only 2.3%. Results are the sum of 2 independent experiments using 192 wells/experiment. (B): Four individual CD133+ cell-derived spheres from (A) were collected and analyzed by flow cytometry. As shown, the percentage of CD133+ cells in the spheres ranged from 54-86% indicating that colonies derived from individual CD133+ STA-ET-8.2 cells contain both CD133+ and CD133- cells.

Prior studies have shown that CD133+ tumor-initiating cells uniquely form and can be expanded as 3-dimensional spheres in non-adherent culture conditions [5,22]. Therefore, we compared sphere-forming capacity of CD133+ and CD133- STA-ET-8.2 cells *in vitro*. Single CD133+ or CD133- cells were independently seeded into ultra low adhesion 96-well plates that favor the proliferation of undifferentiated cells. The number of spheres was scored after 3-4 weeks in culture. Importantly, we observed that the ability of CD133+ cells to generate and proliferate as tumor spheres was 5-fold greater than CD133- cells. Multi-cellular spheres consisting of 500 to 3000 cells (Fig 4A; top panel) grew from 11.4% of individually seeded CD133+ cells. In contrast, spheres were generated from only 2.3% of the single-sorted CD133- cells (Fig 4A). Similar results were seen whether cells were sorted into serum-containing or defined bFGF/EGF neurosphere media (not shown). Importantly, consistent with proliferation assays, we observed no difference in sphere-formation ability between CD133+ and CD133- TC71 cells (not shown).

In normal and malignant neural differentiation, CD133+ stem cells undergo asymmetric cell division to generate both CD133+ and CD133- progeny [5]. To determine if CD133+ STA-ET-8.2 cells are similarly capable of generating both CD133+ and CD133- daughter cells we evaluated clonally derived spheres generated from single CD133+ cells. Individual spheres derived from CD133+ cells were expanded in culture for several passages and then progeny analyzed by flow cytometry. As shown in Fig 4B, CD133+ cell-derived spheres contained both CD133+ and CD133- cells. Thus, these data support the hypothesis that the heterogeneity of CD133 expression in STA-ET-8.2 cell cultures is consequence of asymmetric cell division in which CD133+ cells give rise to both CD133+ and CD133- daughter cells.

### CD133+ STA-ET-8.2 cells preferentially form colonies in soft agar and tumors in immune deficient mice

**Figure 5:**
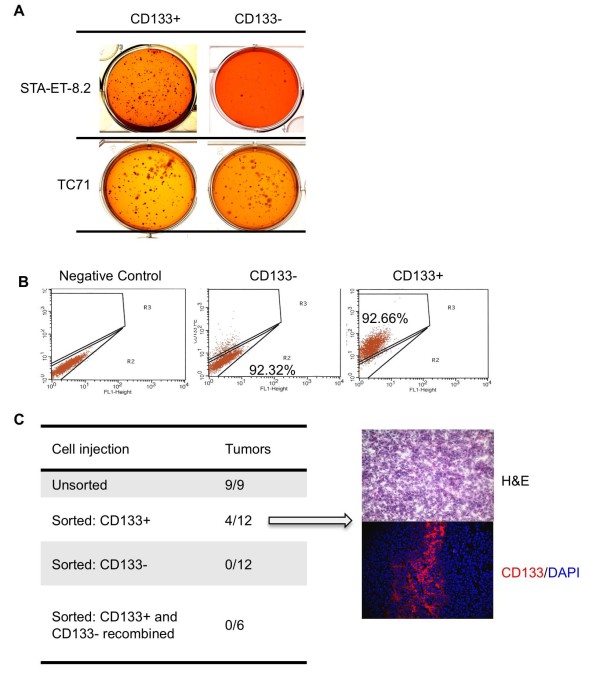
**CD133+ STA-ET-8.2 cells are more tumorigenic than CD133- cells**. (A): CD133+ STA-ET-8.2 cells readily form colonies in soft agar while CD133- STA-ET-8.2 cells form significantly fewer colonies. In contrast, no difference in colony formation is seen in FACS-sorted TC71 cells. Images are representative of 6 wells/experiment from 3 separate experiments. (B): Analysis of CD133 expression in FACS-sorted cells prior to injection confirms significant enrichment of CD133+ cells in CD133+ fraction. (C): Variable tumorigenicity of sorted and unsorted STA-ET-8.2 cells is demonstrated by xenograft assays (left panel). In particular, unsorted STA-ET-8.2 cells reliably form tumors in NOD-SCID mice whereas cells that have been FACS-sorted are highly inefficient. Among sorted populations, only CD133+ cells generated tumors. CD133+ cell-derived xenografts demonstrate the classic small round blue cell ESFT phenotype and contain both CD133+ and CD133- cells (right panel).

By definition cancer stem cells possess tumor-initiating capability [20]. To assess whether CD133+ ESFT cells display enhanced anchorage-independent growth *in vitro *we performed soft agar assays of FACS-sorted CD133+ and CD133- cells. In keeping with their differential growth characteristics in adherent culture, CD133+ STA-ET-8.2 cells displayed dramatically enhanced growth in anchorage-independent conditions compared to CD133- STA-ET-8.2 cells (Fig 5A). In contrast to STA-ET-8.2 cells, and consistent with findings in adherent culture, no differences in colony formation were observed between CD133+ and CD133- cells derived from parental TC71, A4573 or 5838 cell lines (Fig 5A and data not shown). Thus, among the four ESFT cell lines tested, the enhanced clonogenic capacity of CD133+ cells in anchorage-independent conditions was unique to STA-ET-8.2 cells.

Next, we assessed whether increased capacity to generate colonies in soft-agar was associated with increased tumorigenicity *in vivo*. Cells were FACS-sorted into CD133+ and CD133- populations, expanded in culture as independent populations for three to five passages and then equal numbers of cells injected subcutaneously into NOD-SCID mice. Significant enrichment of CD133+ cells and CD133- cells in the respective sorted populations was confirmed immediately prior to cell injection (purity > 90%; Fig 5B). While 4 of 12 mice injected with CD133+ STA-ET-8.2 cells developed a tumor, none of the CD133- cell injections resulted in tumor formation (Fig 5C). Xenograft tumors derived from CD133+ STA-ET-8.2 cells displayed the classic poorly differentiated, homogenous small round blue cell phenotype of ESFT (Fig 5C; right panel). Immunostaining of these xenografts further revealed that the tumors derived from CD133+ cells contained both CD133+ and CD133- cells and the proportion of CD133- cells was significantly greater than the ~7% of contaminating CD133- cells that were present in the tumor cell inoculation (Fig 5C; right panel). In contrast, no differences in tumorigenicity were observed between CD133+ and CD133- TC71 cells: all mice rapidly developed tumors (not shown). Together these data provide preliminary *in vivo *evidence that the tumor-initiating potential of CD133+ STA-ET-8.2 is greater than that of CD133- cells and that CD133+ tumor-initiating cells undergo asymmetric cell division during tumor growth to generate both CD133+ and CD133- progeny. Serial dilution and serial transplantation experiments will be required to define the precise frequency of tumorigenic cells in each of these respective cell populations.

Finally, although the tumorigenicity of CD133+ STA-ET-8.2 cells was enhanced compared to CD133- cells, their capacity to initiate tumors was significantly less than that of unsorted parent cells (Fig 5C). Specifically, although tumors rapidly grew in 9 of 9 mice injected with unsorted STA-ET-8.2 cells, only 4 of 12 mice injected with the same number of CD133+ enriched cells developed a tumor (Fig 5C). This unexpected observation led us to hypothesize that *either *the CD133- cells contribute to the inherent tumorigenicity of the unsorted, parental cell line *or *that the process of FACS-sorting had diminished the relative tumorigenic capacity of all cells. To address this CD133+ and CD133- STA-ET-8.2 cells were collected and injected together. Despite the presence of a 50/50 mix of CD133+ and CD133- cells (which is the homeostatic state of parental, unsorted STA-ET-8.2 cells), no tumors formed in any of the six mice that received this merged cell injection (Fig 5C). These data suggest that the process of FACS-sorting negatively affects the tumor-initiating capacity of STA-ET-8.2 cells irrespective of CD133 expression. Whether MACS-sorting similarly affects the tumor-initiating potential of these cells remains to be evaluated.

### CD133+ STA-ET-8.2 cells are more resistant to chemotherapeutic drugs

**Figure 6:**
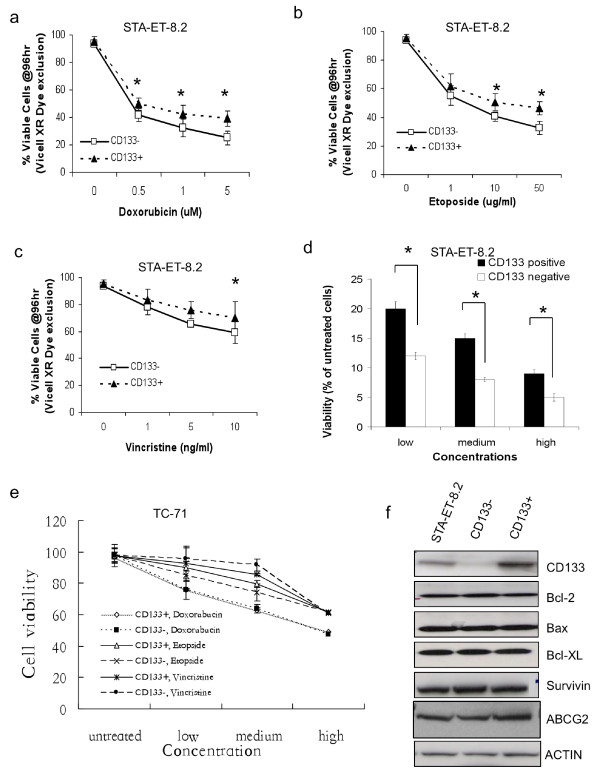
**CD133+ cells are more resistant to chemotherapeutic agents**. STA-ET-8.2 cells were FACS-sorted and CD133+ and CD133- fractions treated with increasing concentrations of (A) Doxorubicin (Doxo), (B) Etoposide (Etop), (C) Vincristine (Vinc) or (D) a combination of all three drugs at high (Doxo 10 μM; Etop 10 ug/ml; Vinc 100 ng/ml), medium (Doxo 2.5 μM; 20 Etop 2.5 ug/ml; Vinc 25 ng/ml) and low (Doxo 0.5 μM; Etop 0.5 ug/ml; Vinc5 ng/ml) concentrations. Viability was assessed by MTS assay after 96 hrs (*p < 0.05) and CD133+ cells displayed increased drug resistance. (E): CD133+ and CD133- TC-71 cells are equally sensitive to Doxo, Etop, and Vinc. (F): Western blot reveals no difference in apoptosis and drug resistance protein expression between unsorted and CD133-sorted STA-ET-8.2 cell fractions.

Cancer stem cells have been reported to be more resistant to chemotherapy [11,23]. We therefore examined the viability of sorted CD133+ and CD133- STA-ET-8.2 cells that were exposed to increasing doses of doxorubicin, etoposide and vincristine alone or in combination for 24-96 hours. As shown (Fig 6A-D), both CD133+ and CD133- STA-ET-8.2 cells displayed a dose-dependent sensitivity to these chemotherapeutic agents. However, the relative resistance of CD133+ cells to drug-induced cell death was significantly greater than for CD133- cells at all three doses (Fig 6A). In contrast, no difference in cell viability was seen between CD133+ and CD133- populations sorted from TC71 (Fig 6E), A4573 or 5838 cell lines (data not shown). Thus, these data demonstrate that CD133 expression enriches for chemo-resistant cells in the STA-ET-8.2 cell line but not in the other ESFT cell lines tested.

Having established that CD133+ STA-ET-8.2 cells display greater drug resistance than autologous CD133- cells, we next sought to investigate the mechanism of this differential sensitivity. FACS-sorted populations were evaluated by western blot for relative expression of proteins involved in apoptosis and multi-drug resistance. No differences between CD133+ and CD133- STA-ET-8.2 cells were observed in the expression of BH3-proteins (Bcl-2, Bcl-XL, Bax), survivin, or the ATP-binding cassette protein ABCG2 (Fig 6F). Similarly, equivalent levels of P-glycoprotein and activated AKT were detected in the sorted and unsorted cells (not shown). Thus, as with many putative cancer stem cells [21], the mechanism of enhanced chemo-resistance in CD133+ STA-ET-8.2 cells remains to be elucidated.

## Discussion

The pentaspan transmembrane glycoprotein CD133, also known as Prominin-1, was originally described as a hematopoietic stem cell marker [24] and subsequently shown to be expressed by a number of progenitor cells, including those of the epithelium where it is expressed on the apical surface [25]. Since the discovery of CD133+ brain tumor stem cells [5], CD133 has been used as a marker for purifying cancer stem cells in other solid tumors, including liver [26], pancreas [27], melanoma[28], prostate [8] and colon [29]. Significantly, a recent study of 8 primary ESFT reported the frequency of CD133+ tumor cells to be 4-8% and functional studies further implicated these cells as putative tumor-initiating cancer stem cells [16].

We evaluated expression of the CD133-encoding gene *PROM1 *in a large cohort of primary ESFT. Consistent with an overall low frequency of CD133+ cells, we found *PROM1 *expression to be extremely low in most cases of ESFT. The absence of detection of *PROM1 *transcript in 5 tumors may be indicative of true negative status or, more likely, a reflection of the RNA having been isolated from very small, closed needle biopsy specimens. Despite the generally low levels of *PROM1 *detection in primary tumors, however, in a significant minority of cases (11 of 48) the transcript was readily detected. Intriguingly, *EWS-ERG *fusion positive cases were significantly over-represented among *PROM1 *expressing tumors. This was corroborated by *in vitro *studies, which also revealed levels of *PROM1/*CD133 to be, in general, higher in *EWS-ERG *than *EWS-FLI1 *cell lines. Whether this difference between tumors with different fusion types is a function of differential effects of the *ETS *fusion partner or a reflection of different cellular origins of *EWS-FLI1 *and *EWS-ERG *tumors is an intriguing question that will require further study.

Significantly, in two cases (one *EWS-FLI1 *and one *EWS-ERG*), high levels of *PROM1 *expression were associated with primary drug-resistant disease. Moreover, in one of these cases the frequency of CD133+ cells increased post-treatment suggesting that the CD133+ fraction contributed to treatment failure. In contrast, however, two other tumors with high levels of *PROM1 *responded well to standard therapy and both patients are long-term, event-free survivors. Unfortunately, frozen tissue was only available for one of the four *PROM1-*high cases so it is not known if high *PROM1 *levels in the other cases were also associated with high levels of glycosylated CD133. It may be that high levels of *PROM1 *are predictive of chemoresistant ESFT but only when accompanied by high expression of the glycosylated CD133 protein. Studies designed to simultaneously evaluate transcript expression levels as well as glycosylated and non-glycosylated CD133 protein expression in fresh frozen ESFT specimens are necessary to address this issue. Thus, expression of *PROM1 *in ESFT is highly variable and the potential clinical significance of high level *PROM1 *transcript expression requires further evaluation in large, prospective studies.

The existence of discrete populations of tumor-initiating cells within established cultures indicates that even cell lines that have been maintained for many years in culture can retain cellular hierarchies in which tumorigenic stem cells give rise to less tumorigenic progeny [15,30-32]. Functional studies of CD133+ and CD133- fractions derived from ESFT cell lines demonstrated significant heterogeneity in their biologic properties. Specifically, although CD133+ cells could be isolated from all ESFT cell lines, only CD133+ cells isolated from the STA-ET-8.2 cell line displayed evidence of stem cell characteristics and chemo-resistance. In contrast, we could discern no phenotypic or functional differences between CD133+ and CD133- cells derived from other ESFT cell lines. Whether this inconsistency is a result of genetic evolution *in vitro *or a true reflection of variable significance of CD133 expression in the original tumors remains to be determined.

Importantly, recent studies have challenged the utility of CD133 as a single marker of tumor-initiating cell populations. CD133- tumor cells derived from some primary tumors and cell lines possess self-renewal and tumor-initiating potential even when injected at very low numbers [22,32-34]. These conflicting data, when combined with the uncertain biological role of CD133, highlight the need for additional distinguishing markers that are directly involved in maintaining the functional properties of the putative cancer stem cell population [35]. Indeed, the variability in *PROM1 *expression in primary ESFT combined with the inconsistent biologic properties of CD133+ ESFT cells in culture suggest that CD133 expression alone will be insufficient to isolate drug-resistant cancer stem cells in ESFT. Nevertheless, our data indicate that *PROM1/*CD133 expression may be a useful marker of increased chemoresistance in at least some cases of primary ESFT and that the STA-ET-8.2 cell line will be a useful tool to study the biology of these cells in the laboratory.

## Conclusions

Drug-resistant disease at diagnosis or at relapse remains a major cause of mortality among patients diagnosed with ESFT. An improved understanding of the mechanisms of drug-resistance and biomarkers that can prospectively identify resistant tumors are desperately needed. Our studies indicate that in some cases of ESFT over-expression of the *PROM1 *transcript is associated with primary drug-resistant disease. Future studies of prospectively acquired primary tumors are now required to definitively address the clinical significance of *PROM1 *expression and its relationship to drug-resistant CD133+ cells in ESFT.

## Competing interests

The authors declare that they have no competing interests.

## Authors' contributions

XJ, YG, DR, CC, carried out the *in vitro *studies. DD and LH carried out the *in vivo *studies. HK and TJT participated in the design of the study and provided tumor and cell samples. XJ and ERL conceived of the study and participated in its design and coordination. XJ and ERL wrote the manuscript. All authors read and approved the final manuscript.

## Pre-publication history

The pre-publication history for this paper can be accessed here:

http://www.biomedcentral.com/1471-2407/10/116/prepub
